# Hard to Swallow: Tongue Avulsion Secondary to Hypocortisolism Posing a Difficult Airway

**DOI:** 10.7759/cureus.108562

**Published:** 2026-05-09

**Authors:** João Batalha Serafim, Madalena Mariano Pereira, Francisco Valente, Inês Tomé

**Affiliations:** 1 Anesthesiology, Unidade Local de Saúde de São José, Lisbon, PRT

**Keywords:** airway bleeding, difficult airway management, hypocortisolism, maxillofacial trauma, tongue avulsion

## Abstract

Maxillofacial trauma involving tongue injuries poses a significant challenge for airway management due to active bleeding, anatomical distortion, and the risk of obstruction and aspiration.

We report the case of a 37-year-old man who presented with a self-inflicted tongue avulsion, likely in the context of an epileptic seizure. The patient was orotracheally intubated and transferred by helicopter under sedation and mechanical ventilation to a tertiary center, where the surgical team requested nasotracheal intubation to facilitate intraoral access. However, active bleeding and avulsion of tongue segments posed a high risk of complications, so it was decided to reposition the existing orotracheal tube under continuous suction and videolaryngoscopic guidance.

This case highlights the importance of tailoring anesthetic and airway strategies to the specific anatomical and surgical context, underscoring the need for targeted simulation training in maxillofacial airway management.

## Introduction

Airway management in patients with maxillofacial trauma is particularly challenging for anesthesiologists. While anatomical distortion and the risk of obstruction are significant, active hemorrhage remains the primary concern [1]. Heavy bleeding can significantly hinder the view of usual anatomical landmarks and make direct laryngoscopy either very challenging or impossible. Moreover, blood accumulation in the pharynx increases the risk of pulmonary aspiration, requiring continuous suction [2,3]. In cases of extensive tongue lacerations, abnormal tongue mobility further worsens exposure difficulties during intubation [1]. Early adoption of videolaryngoscopy devices - which allow better angulation and indirect visualization of the vocal cords - combined with high-flow suction systems is recommended [4,5]. In such cases, preparation for intubation must include well-defined contingency plans to ensure patient safety and successful intubation.

This case report was previously presented as a poster at the 19th World Congress of Anaesthesiologists on April 18, 2026. 

## Case presentation

A 37-year-old male with a history of heavy tobacco and alcohol use presented to a district hospital emergency department following a ground-level fall. While in the waiting room, he became agitated, diaphoretic, and confused, with active oral bleeding and repetitive chewing movements of his own tongue. No tonic-clonic seizure was observed. On examination, there was an avulsion of the anterior half of the tongue, with several devitalized tissue segments.

He was transferred by helicopter to our tertiary hospital for assessment by the maxillofacial surgery team. To secure the airway, he was transported under mechanical ventilation with continuous infusions of propofol and fentanyl following difficult orotracheal intubation performed using a videolaryngoscope and an 8.0 mm cuffed tube after rapid-sequence induction. During the transfer, myoclonic movements were observed.

**Figure 1:**
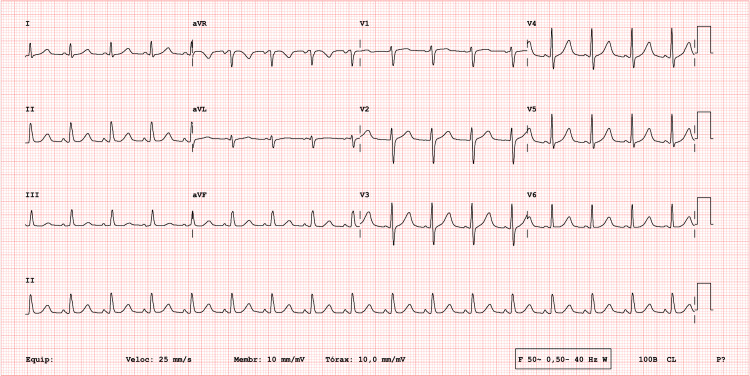
Preoperative 12-lead electrocardiogram

Upon arrival at our hospital, the surgical team assessed the patient and decided to proceed with surgical exploration and primary closure. Preoperative workup, including blood tests and an electrocardiogram (ECG), was performed. The ECG showed sinus rhythm at 100 beats per minute and a borderline prolonged QT interval (451 ms), with no other significant abnormalities (Figure 1). Blood test results, summarized in Table 1, were notable for hyponatremia (128 mmol/L), reflecting partial correction from the initial admission value of 118 mmol/L at the referring hospital. Accordingly, appropriate clinical adjustments were made before surgery. 

**Table 1:** Preoperative blood analysis results aPTT: Activated Partial Thromboplastin Time; INR: International Normalized Ratio; PT: Prothrombin Time

Parameter	Results	Reference range
Hemoglobin	12.9 g/dL	13.0 - 17.0 g/dL
Leukocytes	16.5 x 10^9^/L	4.5 - 11.0 x 10^9^/L
Neutrophils	15.5 x 10^9^/L	2.0 - 8.5 x 10^9^/L
Platelets	112 x 10^9^/L	150 - 450 x 10^9^/L
PT	9.7 seconds	9.4 - 12.5 seconds
INR	0.81	0.80 - 1.20
aPTT	25.2 seconds	25.1 - 36.5 seconds
Fibrinogen	2.4 g/L	2.0 - 4.0 g/L
Creatinine	0.96 mg/dL	0.67 - 1.17 mg/dL
Sodium	128 mmol/L	136 - 145 mmol/L
Potassium	3.1 mmol/L	3.5 - 5.1 mmol/L
Chloride	86 mmol/L	98 - 107 mmol/L
Calcium	8.1 mg/dL	8.6 - 10.0 mg/dL

**Figure 2:**
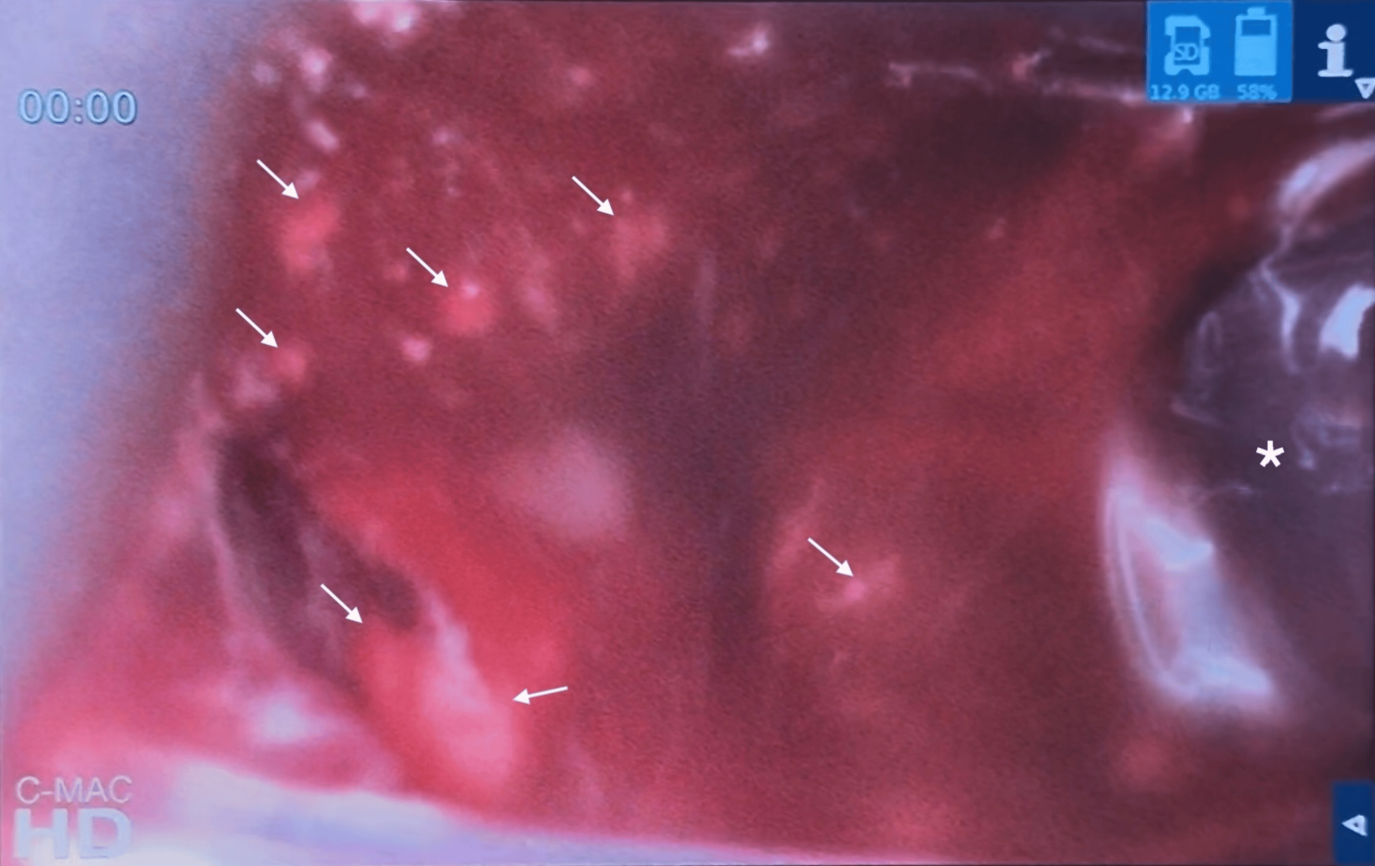
Video frame of the C-MAC® visualization before suctioning The image shows a severely soiled airway characterized by field-obscuring hemorrhage and dispersed devitalized tongue debris. The arrows point to segments of avulsed tongue tissue. The asterisk (*) identifies the proximal portion of the orotracheal tube.

**Figure 3:**
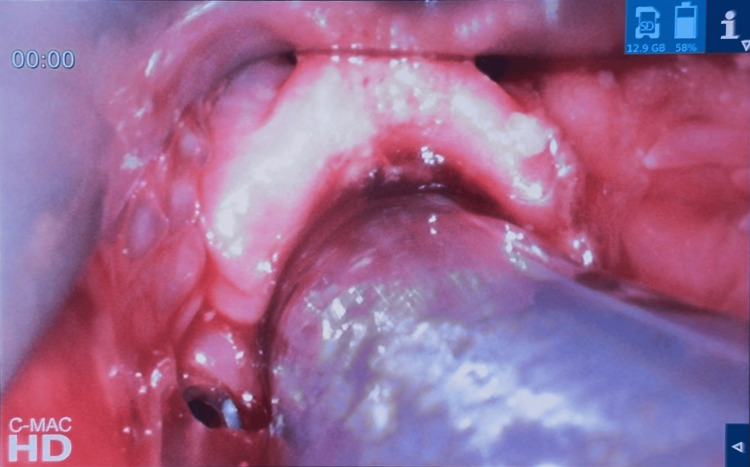
C‑MAC® view of the epiglottis and orotracheal tube following suctioning and blade advancement

To proceed with the intervention, the surgical team requested removal of the orotracheal tube to improve access to the oral cavity and tongue. As such, nasotracheal intubation was considered contingent on prior visualization with the C‑MAC® D-Blade videolaryngoscope (Karl Storz, Tuttlingen, Germany). Blade insertion was challenging due to anatomical distortion from avulsion of multiple tongue segments. Visualization revealed pooled blood and dispersed fragments of devitalized tongue tissue (Figure 2). Continuous suction cleared the field sufficiently to advance the blade and identify the epiglottis, confirming that the orotracheal tube remained properly positioned (Figure 3). Given the high risk of further tongue avulsion, increased bleeding, tissue fragment displacement, and aspiration during a nasotracheal intubation attempt - and since the airway was already secured and the patient adequately ventilated - it was agreed with the surgical team to maintain the orotracheal tube. The tube was then repositioned laterally toward the right commissure with Magill forceps under continuous suction and videolaryngoscopic guidance. 

The revised tube position enabled completion of the surgical procedure, during which the tongue avulsion injury was repaired with primary suturing. The patient was admitted to an intensive care unit (ICU), where he remained for 19 days. He was extubated on ICU day 12, and a tracheotomy was performed due to post-intubation laryngeal edema. He was discharged on hospital day 23 for ongoing care at his local hospital. The patient underwent tracheotomy decannulation on postoperative day 28 and was referred for follow-up at the otorhinolaryngology outpatient clinic. Further workup attributed the tongue trauma to a focal seizure with automatisms occurring in the setting of severe hyponatremia (serum sodium level on admission: 118 mmol/L), secondary to abrupt withdrawal of corticosteroid therapy for low back pain, resulting in severe hypocortisolism. 

## Discussion

Tongue injuries occasionally present in the emergency department and can result from a variety of etiologies, including epileptic seizures, self-inflicted trauma, facial injuries, or iatrogenic trauma during intubation [1]. While direct pressure typically achieves primary hemostasis, this method might be insufficient in some cases, especially when the lingual artery is affected or in individuals with coagulopathies [2,3].

To our knowledge, three case reports have described the management of traumatic tongue avulsion or near-amputation using different airway approaches [6-8]. Hernández-Méndez et al. reported emergent nasotracheal intubation and primary layered suturing for a partial avulsion [6]. Egozi et al. performed tracheotomy followed by microvascular replantation for a near-total tongue amputation [7]. A report of self-inflicted segmental tongue amputation described emergent tracheotomy and microsurgical replantation [8].

Managing the airway in patients with tongue trauma can be difficult, even for experienced staff. Primarily, active bleeding may obscure the visual field, so it is crucial to ensure that high-flow suction is readily available and to apply topical hemostatic agents such as epinephrine, aminocaproic acid, and tranexamic acid. Furthermore, injuries to the oral cavity and adjacent tissues can deform the anatomical references typically used during intubation. Moreover, in patients with concomitant cervical spine injuries who are immobilized in a cervical collar, the neck must be maintained in a neutral position, precluding cervical hyperextension and further complicating laryngoscopy. Therefore, in the context of maxillofacial trauma, nasotracheal intubation - which is already technically demanding under routine circumstances - can become significantly more difficult [9]. For all these reasons, when managing these airways, it is essential to have adjunct devices readily available, such as flexible tracheal introducers (e.g., bougies), videolaryngoscopes, and flexible bronchoscopes, and to prioritize awake intubation with spontaneous respiratory drive to facilitate anticipated difficult intubation and avoid complications. Additionally, personnel should be prepared to establish a surgical airway as a backup plan, and the necessary equipment must be immediately available in the room if initial intubation attempts fail [2,4,9].

In this rare case of anterior tongue avulsion, the surgical team requested the exchange of the preexisting orotracheal tube for a nasotracheal tube to improve surgical access. However, attempting nasotracheal intubation in the setting of active hemorrhage and anatomical distortion - even with videolaryngoscopic assistance - carried a significant risk of failed intubation and airway compromise due to potential avulsion of the residual tongue tissue, exacerbation of bleeding, displacement of tissue fragments, and aspiration of blood and debris. Consequently, the strategy prioritized maintaining the already secured and ventilatable airway over the anticipated challenges of high-risk nasotracheal intubation. This decision is consistent with international guidelines, which emphasize avoiding multiple intubation attempts [4,5]. Continuous suction and videolaryngoscopy proved essential for secure airway evaluation. This shared real-time visualization also facilitated multidisciplinary consensus, enabling the anesthetic and surgical teams to reach an informed, deliberate decision. The lateral repositioning of the orotracheal tube not only ensured optimal surgical exposure but also maintained ventilation without compromise. If surgical access had not been attainable under these conditions, the next step would have been to perform a tracheotomy, since an alternative such as submental transposition of the tube would not have been feasible given the circumstances.

## Conclusions

In the context of traumatic tongue avulsions, airway management is a critical intersection of anatomical disruption, hemorrhage, and imminent aspiration risk. The risk of failed intubation mandates videolaryngoscopy and continuous high-flow suction as a standard of care, with a low threshold for establishing a front-of-neck airway. This case highlights the importance of critical decision-making when surgical and anatomical factors conflict with airway safety. Integrating multidisciplinary consensus, targeted simulation training, and structured algorithms is essential to enhance clinical readiness and optimize outcomes in complex maxillofacial trauma.
